# Duodenal Graft Perforation after Simultaneous Pancreas-Kidney Transplantation

**DOI:** 10.1155/2017/5681251

**Published:** 2017-04-05

**Authors:** Akihito Sannomiya, Ichiro Nakajima, Yuichi Ogawa, Kotaro Kai, Ichiro Koyama, Shohei Fuchinoue

**Affiliations:** Department of Surgery, Kidney Center, Tokyo Women's Medical University, Tokyo, Japan

## Abstract

A 45-year-old woman with type 1 diabetes and chronic renal failure on dialysis underwent simultaneous pancreas-kidney transplantation from a brain dead donor. On postoperative day 15, acute generalized peritonitis was diagnosed and emergency laparotomy was performed. Perforation of the donor duodenum was found, which had apparently resulted from duodenal compression by the tip of the intestinal fistula tube placed for decompression. The perforation was sutured and the intestinal fistula tube was exchanged. Following this, perforation repeatedly recurred at the same site and open repair at laparotomy was required a total of four times. The fourth operation involved both suturing the perforation and covering it with ileum, after which there was no further recurrence. The patient was discharged on posttransplantation day 219, with the pancreas and kidney grafts both functioning well. This report presents a rare complication of simultaneous pancreas-kidney transplantation.

## 1. Introduction 

In Japan, the Organ Transplant Act was revised in July 2010, leading to an increase of patients undergoing simultaneous pancreas and kidney transplantation from brain dead donors, which has been established as surgical treatment for diabetes [1]. Common complications of pancreatic transplantation include graft thrombosis, graft pancreatitis, and rejection, but perforation of the associated duodenal graft is rare [2]. Nath et al. described late anastomotic leaks with bladder drainage as not uncommon [3]. Here we report a patient who required open repair four times for perforation of the donor duodenum due to compression by an intestinal tube after simultaneous pancreas-kidney transplantation. Eventually, both grafts were preserved and remained functional.

## 2. Case Report

A 45-year-old woman was diagnosed with type I diabetes at the age of 16 years and hyperthyroidism at age 20, as well as acute pancreatitis at 22 years, caesarean section at 30 years, and gastric ulcer at the age of 38.

She received insulin therapy for diabetes. At the age of 33 years, she started hemodialysis due to chronic renal failure caused by diabetic nephropathy. Hypoglycemic episodes were frequent. She was admitted to our department for simultaneous pancreas and kidney transplantation from a brain dead donor (a 39-year-old man with cerebral hemorrhage).

On admission, she was 156 cm tall, weighed 54.0 kg, and had a body mass index of 22.2 kg/m^2^. Her total insulin dose was 27 units/day (ultra-long-acting insulin: 10 units in the morning, ultrarapid insulin: 17 units). Hemoglobin A1c (HbA1c) was 8.2% (National Glycohemoglobin Standardization Program value), glycoalbumin was 34.1%, and C-peptide was <0.0099 nmol/L. In addition, anti-insulin antibodies were <0.4 kU/L, antiglutamic acid decarboxylase antibodies were 6.5 kU/L, and anti-islet antigen-2 antibodies were <0.4 kU/L.

*Surgery*. The pancreas was transplanted into the right iliac fossa and the kidney was transplanted into the left iliac fossa. To drain exocrine secretions, a Y-limb was created at the terminal ileum by the Roux-en-Y method and was anastomosed side-to-side to the donor duodenum following vascular anastomosis. The donor portal vein was anastomosed to the recipient's right external iliac vein, and the celiac artery and a Carrel patch extending from the superior mesenteric artery were each anastomosed to the recipient's right external iliac artery. The pancreatic graft was placed inside the abdominal cavity, as it was large relative to the recipient. Since the donor duodenum was distended, an intestinal fistula tube was placed for decompression of the intestinal tract. A 16 Fr Salem sump tube was inserted from the distal part of the ileal Y-limb, and the tip was advanced beyond the anastomosis and fixed inside the donor duodenum.

The total ischemic time was 9 hours and 24 minutes for the pancreas and 10 hours and 54 minutes for the kidney. Blood loss was 370 g. 

**Figure 1:**
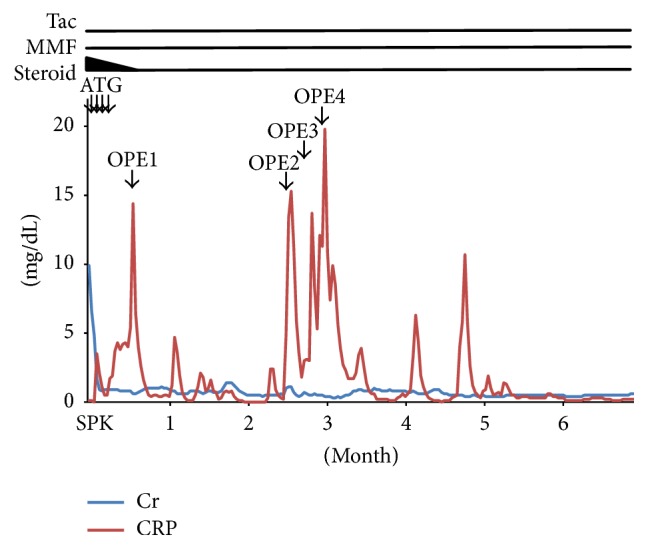
Postoperative course. The pancreas graft was salvaged by four laparotomies to repair duodenal perforation after pancreas-kidney transplantation.

*Postoperative Course*. The postoperative course is outlined in Figure 1. The patient had an adequate urine output after transplantation, so dialysis was stopped. Insulin therapy was also discontinued because a decrease of blood glucose was observed. Immunosuppressive therapy was initiated with rabbit antithymocyte globulin (1.5 mg/kg for 4 days), in addition to tacrolimus, mycophenolate mofetil, and steroids. Antithrombotic therapy was commenced on postoperative day 1 with continuous infusion of heparin at 2500 units/day (50 units/kg/day). Warfarin was started on postoperative day 8 and heparin was stopped on postoperative day 11. Valganciclovir was administered to prevent cytomegalovirus (CMV) infection, starting from one week after transplantation. Administration of GFO (a powdered food supplement containing glutamine, fiber, and oligosaccharides suggested to improve intestinal health) was on postoperative day 5. The intestinal tube was allowed to drain spontaneously without negative pressure.

**Figure 2:**
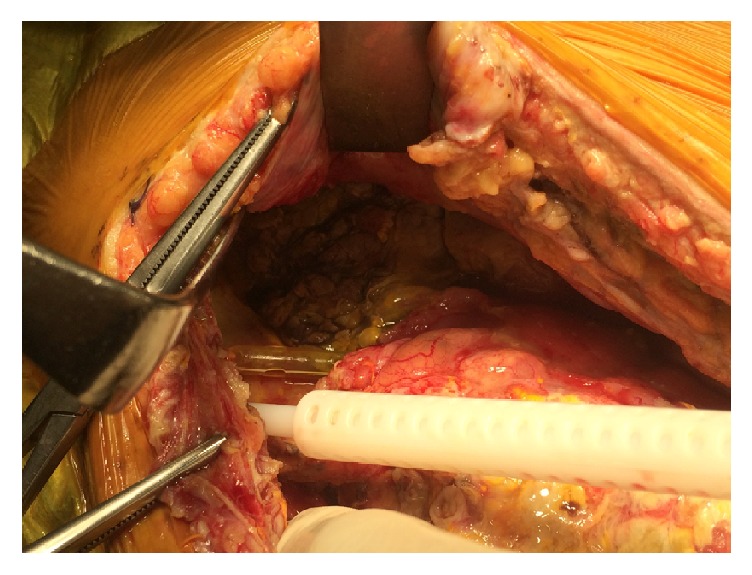
Postoperative day 15. Perforation of donor duodenum appeared to be due to the tip of the intestinal tube penetrating the wall.

On postoperative day 14, fever (temperature ≥ 38°C) and abdominal pain developed. On postoperative day 15, there were signs of peritoneal irritation. Abdominal CT revealed ascites and free intraperitoneal gas. Generalized peritonitis due to gastrointestinal perforation near the pancreatic graft was diagnosed and emergency laparotomy was performed. The previous surgical wound in the right lower abdomen was reopened. The abdominal cavity contained intestinal fluid, and perforation of the donor duodenum was confirmed (Figure 2). Perforation appeared to be due to the tip of the intestinal tube penetrating the duodenal wall. Partial resection of the duodenal segment including the site of perforation was considered but was judged to be difficult due to severe inflammation and edema of the donor duodenum, as well as the close proximity of the perforation to the anastomotic site. Therefore, the perforation was sutured and the intestinal tube was exchanged for a 9 Fr jejunostomy catheter (3 mm outer diameter) via the same route as the Salem sump tube.

The initial postoperative course was favorable and oral intake was started on day 17 after reoperation, but bowel obstruction developed five days later. It seemed to be caused by adhesions and resolved after decompression with a gastric tube. Oral intake was started again on day 25 following removal of the gastric tube, but fever occurred after six days. Peritonitis was diagnosed two days later and emergency laparotomy was performed via the previous surgical wound (second reoperation). Since there was reperforation of the donor duodenum at the previous perforation site, a 3.5 mm silicon tube (Lilia drain) was inserted through the perforation into the duodenum as a second intestinal tube and was anchored with a purse-string suture.

On day 7 after the second reoperation, peritonitis recurred and another emergency laparotomy was performed via the same surgical wound in the right lower abdomen (third reoperation). Leakage of pancreatic juice was observed from the site where the silicon tube had been inserted into the donor duodenum. This tube was removed and omental patch repair was performed. The intestinal tube was replaced by a new 9 Fr jejunostomy catheter (3 mm outer diameter).

On day 5 after the third reoperation, peritonitis was diagnosed again and yet another emergency laparotomy was performed (fourth reoperation). This time, a midline abdominal incision was made, in addition to reopening the wound in the right lower abdomen. It was found that pancreatic juice was leaking from the edge of the omental patch. The patch was completely removed, and, after extensively peeling off the adhesions around the donor duodenal perforation, it was closed with a simple two-layer suture. Then adhesions were peeled off the terminal ileum and fixed over the sutured duodenal perforation as a small intestinal patch. Damage to the ascending colon, which had probably occurred when adhesions were being removed, was also managed by two-layer suture closure.

GFO was started on day 45 after the fourth reoperation. Around this time, neutropenia and thrombocytopenia developed but resolved after discontinuation of valganciclovir and proton pump inhibitor therapy. On day 69 after the fourth reoperation, a nonfat digested liquid diet was started. Food intake was commenced on day 78, followed by slow transition from a liquid to a solid diet, and central venous nutrition was stopped on day 89. She was discharged on day 121 after the last operation (posttransplantation day 219). At discharge, serum creatinine was 0.61 mg/dL the estimated glomerular filtration rate was 82.1 mL/min/1.73 m^2^, urinary protein was negative, C-peptide was 0.53 nmol/L, fasting blood glucose was 116 mg/dL, and HbA1c was 5.0%. The total insulin dose at discharge was 11 units/day (7 units of Novolin R short-acting insulin and 4 units of Apidra ultra-rapid-acting insulin). Two months after discharge, she was readmitted and the intestinal tube was removed uneventfully.

## 3. Discussion

As of July 2016, our hospital has performed a total of 60 pancreatic transplantations, including 11 from nonbeating heart donors and 49 from brain dead donors. However, this is our first experience of a patient with perforation of the donor duodenum. Our literature search revealed only 8 reported cases worldwide of donor duodenum perforation after pancreatic transplantation (Table 1) [4–11].

In these 9 patients, including our case, perforation occurred from 14 days to 27 months postoperatively. The cause of perforation was rejection in three patients, CMV infection in two patients, and donor duodenal ulcer, intestinal obstruction by an internal hernia, ischemic necrosis at the anastomosis, and compression necrosis due to the tip of an intestinal decompression tube in one patient each. Although we did not perform histopathological examination, rejection was considered unlikely in the present case because function of the transplanted kidney and pancreas was satisfactory. Moreover, CMV prophylaxis was initiated at an early stage, and the CMV antigenemia test and quantitative polymerase chain reaction for CMV-DNA were both negative, so CMV infection was excluded. Ischemic necrosis was also excluded from the macroscopic appearance of the donor duodenum. Accordingly, we concluded that perforation was due to sustained compression of the duodenal wall by the tip of the intestinal tube, which was also suggested by the intraoperative findings. The tube was initially placed away from the duodenal wall, but the donor duodenum may have shortened as edema resolved, allowing the tip of the catheter to compress the duodenal wall and cause perforation. The efficacy of the intestinal tube in pancreas transplant is not established. We opted for the tube insertion because of distention of duodenum but these series of perforation might be avoided without the insertion.

Among the previously reported cases, surgical treatment included simple closure of the perforation in three patients, removal of the donor duodenum in two patients, and total graft removal in three patients. Thus, pancreatic graft preservation was possible in six patients, including our patient. It was reported that 70% of patients who developed intra-abdominal infection after pancreatic transplantation required graft removal [12]. In Japan, the number of brain dead donors is low compared with that in Europe or the United States, so it is desirable to preserve grafts whenever possible. However, patient survival must retain priority, and we should not hesitate to remove the graft if required. In our patient, intestinal perforation occurred in the relatively early postoperative period, and placement of the peritoneal drain and intestinal decompression tube may have prevented the rapid progression of peritonitis. Also, our patient's general condition did not deteriorate despite multiple laparotomies and no other complications developed, which were major factors in allowing successful graft preservation.

## 4. Conclusion 

In the present patient, preservation of the grafted pancreas was achieved by performing laparotomy four times to repair perforation of the donor duodenum causing acute generalized peritonitis, which resulted from the tip of an intestinal tube compressing the duodenal wall after pancreas transplantation. We reported this case because it represents a rare and interesting complication of pancreatic transplantation.

## Figures and Tables

**Table 1:** Reported cases of donor duodenal perforation after pancreatic transplantation. Pancreatic graft preservation was possible in six patients, including our patient.

Case number	Author	Age	Gender	Complaint	Exocrine drainage	Cause of perforation	Interval from surgery to perforation	Operation
(1)	Gruessner et al. [4]	30	M	Abdominal pain	Enteric	Rejection	27 months	Graft duodenectomy
(2)	Schleibner et al. [5]	33	M	Abdominal pain	Bladder	Simple ulcer	5 months	Direct closure
(3)	Stephanian et al. [6]	32	F	Abdominal pain	Bladder	Cmv duodenitis	18 months	Graft duodenectomy
(4)	Ester et al. [7]	47	F	Hematuria	Bladder	Rejection	1.5 months	Direct closure
(5)	Lee et al. [8]	30	F	Hematuria	Bladder	CMV duodenitis	2 months	Graft pancreatectomy
(6)	Fumimoto et al. [9]	37	F	Abdominal pain	Enteric	Internal hernia	13 months	Graft pancreatectomy
(7)	Miyagi et al. [10]	60	M	Melena	Enteric	Rejection	0.5 months	Graft pancreatectomy
(8)	Yamamoto et al. [11]	45	F	Abdominal pain	Enteric	Avascular necrosis	5 months	Direct closure
(9)	Present case	45	F	Abdominal pain	Enteric	Pressure necrosis	0.5 months	Direct closure

## References

[1] Egawa H., Tanabe K., Fukushima N., Date H., Sugitani A., Haga H. (2012). Current status of organ transplantation in Japan. *American Journal of Transplantation*.

[2] Troppmann C. (2010). Complications after pancreas transplantation. *Current Opinion in Organ Transplantation*.

[3] Nath D. S., Gruessner A., Kandaswamy R., Gruessner R. W., Sutherland D. E. R., Humar A. (2005). Late anastomotic leaks in pancreas transplant recipients—clinical characteristics and predisposing factors. *Clinical Transplantation*.

[4] Gruessner R. W., Manivel C., Dunn D. L., Sutherland D. E. (1991). Pancreaticoduodenal transplantation with enteric drainage following native total pancreatectomy for chronic pancreatitis: a case report. *Pancreas*.

[5] Schleibner S., Theodorakis J., Illner W. D., Leitl F., Abendroth D., Land W. (1992). Ulcer perforation in the grafted duodenal segment following pancreatic transplantation—a case report. *Transplantation Proceedings*.

[6] Stephanian E., Gruessner R. W. G., Brayman K. L. (1992). Conversion of exocrine secretions from bladder to enteric drainage in recipients of whole pancreaticoduodenal transplants. *Annals of Surgery*.

[7] Esterl R. M., Stratta R. J., Taylor R. J., Radio S. J. (1995). Rejection with duodenal rupture after solitary pancreas transplantation: an unusual cause of severe hematuria. *Clinical Transplantation*.

[8] Lee H. K., Chung D. H., Jung J. (2000). Three cases of pancreas allograft dysfunction. *Journal of Korean Medical Science*.

[9] Fumimoto Y., Tanemura M., Hoshida Y., Nishida T., Sawa Y., Ito T. (2008). Graft duodenal perforation due to internal hernia after simultaneous pancreas-kidney transplantation: report of a case. *Case Reports in Gastroenterology*.

[10] Miyagi S., Sekiguchi S., Kawagishi N. (2011). Nonmarginal-donor duodenal ulcers caused by rejection after simultaneous pancreas and kidney transplantation: a case report. *Transplantation Proceedings*.

[11] Yamamoto T., Narumi S., Okada M. (2015). Delayed graft duodenal perforation after simultaneous pancreas-kidney transplantation. *Japanese Journal of Gastroenterological Surgery*.

[12] Gruessner R. W. G., Sutherland D. E. R., Troppmann C. (1997). The surgical risk of pancreas transplantation in the cyclosporine era: an overview. *Journal of the American College of Surgeons*.

